# Reviewed article: Santos DS, Viana PVS, Basta PC. Observational study of the coverage rate and survival analysis of COVID-19 vaccination among Indigenous people in Roraima, 2021-2023. Epidemiol Serv Saude. 2025:34;e20240762

**DOI:** 10.1590/S2237-96222025v34e20240762.c

**Published:** 2025-10-24

**Authors:** Maria Paula do Amaral Zaitune

**Affiliations:** 1 Universidade de Brasília, Brasília, DF, Brazil

## First round comments

O manuscrito aborda um tema de relevância significativa para a área, contribuindo com resultados importantes para o campo de estudo. No entanto, para que o trabalho atinja seu potencial máximo, recomendamos que os autores realizem todas as adequações apontadas pelos revisores, as quais visam aprimorar a qualidade e a clareza do texto.

Além disso, sugerimos uma revisão cuidadosa do texto em relação à língua portuguesa, com foco especial na clareza e na coesão, principalmente nas seções de metodologia e apresentação dos resultados. É fundamental que sejam detalhadas e precisas, garantindo que os procedimentos adotados e os resultados obtidos sejam compreendidos de forma inequívoca pelos leitores.

As tabelas também requerem atenção especial. Recomendamos que sejam revisadas para garantir que estejam corretamente formatadas, autoexplicativas e com clareza nas informações apresentadas que facilitem a interpretação dos resultados.

Por fim, encorajamos os autores a aproveitarem as sugestões dos revisores para aprimorar a qualidade geral do manuscrito, tornando-o mais robusto e alinhado aos padrões acadêmicos esperados.

Recommendation: Minor revision

## Second round comments

As recomendações dos revisores foram acatadas e as devidas alterações realizadas, sendo todas indicadas em carta e destacadas no texto. Há apenas um apontamento pois, embora relatem a mudança de título, a nova versão em pdf não reflete esta mudança.

Proposta: “Cobertura vacinal contra a covid-19 em indígenas de um estado da região Amazônica brasileira: uma análise de sobrevivência de 2021 a 2023”.

Aparece como: “Estudo observacional da taxa de cobertura e sobrevivência vacinal do novo coronavírus nos indígenas de Roraima entre 2021 a 2023”

Recommendation: Major revision

